# Macrophage-specific targeting of histone demethylases with small-molecule inhibitors suppresses inflammatory response *in vivo*

**DOI:** 10.1016/j.jbc.2026.113315

**Published:** 2026-07-06

**Authors:** Niranjana Natarajan, Thaybah Ahmed, Balaji Govindaswamy, Devika S. Manickam, Jishnu Das, Kabirul Islam, Partha Dutta

**Affiliations:** 1 Division of Cardiology, Department of Medicine, Pittsburgh Heart, Lung, Blood, and Vascular Medicine Institute, University of Pittsburgh School of Medicine, University of Pittsburgh Medical Center, Pittsburgh, Pennsylvania, USA; 2 Department of Neurology, McGovern Medical School, UT Health Houston, Houston, Texas, USA; 3 Department of Immunology, University of Pittsburgh, Pittsburgh, Pennsylvania, USA; 4 Department of Immunology, Center for Systems Immunology, Pittsburgh, Pennsylvania, USA; 5 Department of Chemistry, University of Pittsburgh, Pittsburgh, Pennsylvania, USA; 6 Department of Bioengineering, Swanson School of Engineering, University of Pittsburgh, Pittsburgh, Pennsylvania, USA; 7 Veterans Affairs Pittsburgh Healthcare System, Pittsburgh, Pennsylvania, USA

**Keywords:** epigenetic modifications, histone demethylases, inflammation, innate immune cells, macrophages

## Abstract

Macrophages are versatile immune cells, with the ability to respond to varied intrinsic and extrinsic cues and transition between inflammatory and proreparative phenotypes. A complex network of epigenetic processes, such as DNA methylation, histone methylation, and acetylation, plays key roles in modulating macrophage polarization and inflammatory gene expression. Transcriptional analysis in patients with respiratory failure, long COVID-19, and influenza revealed an augmented expression of chromatin modifiers, including histone demethylases, broadly defined as lysine demethylases (KDMs), in lung macrophages. To investigate the role of KDMs in inflammation, we screened a panel of small-molecule inhibitors of chromatin modifiers for their efficacy in inducing anti-inflammatory macrophage polarization *in vitro*. We demonstrate that pretreatment with the broad-spectrum KDM inhibitor n-octyl-IOX1 and KDM5-specific inhibitor PB-IT resulted in a significant decrease of lipopolysaccharide (LPS)-induced expression of the inflammatory genes Il1b, Il6, Tnfa, and iNos in bone marrow–derived macrophages. Subsequent RNA sequencing and CUT&RUN analyses revealed that LPS activation led to distinct transcriptomic and epigenomic alterations, including expression of master transcription factors BLIMP-1 and GFI1, whereas n-octyl-IOX1 and PB-IT treatments rewired these regulatory networks, thereby impeding inflammatory gene expression. To further probe the merit of KDM inhibition in perturbing macrophage-mediated inflammation *in vivo*, we delivered n-octyl-IOX1 selectively to macrophages in mice using cell-specific, targeted lipidoid nanoparticles. n-octyl-IOX1 encapsulated nanoparticles significantly diminished LPS-mediated peritoneal macrophage expansion and inflammatory gene expression in this cell population, underscoring the importance of macrophage-specific targeting of KDMs with small-molecule inhibitors in inflammatory disease.

Macrophages, a component of the innate immune system, are key players in inflammatory response, and tissue repair and homeostasis (1, 2, 3). In addition to detection and phagocytosis of foreign materials like bacteria, a crucial function of macrophages is to clear cellular debris and apoptotic cells for inflammation resolution (2, 3). The functional diversity of macrophages stems from their phenotypic plasticity, wherein intrinsic and extrinsic cues modulate differentiation of resting macrophages to adopt a specific state, a process commonly known as macrophage polarization (4, 5, 6). A simplified classification of macrophage polarization describes classically activated pro-inflammatory macrophages and alternatively activated reparative macrophages (5, 7, 8). Classical macrophage activation is induced by microbial products that are Toll-like receptor (TLR) ligands such as lipopolysaccharide (LPS) and interferon-γ (6, 9). After classical activation, macrophages secrete inflammatory cytokines (IL-1, IL-12, TNF⍺, *etc.*), reactive oxygen species, and nitric oxide, recruit additional leukocytes, and mediate host defense by clearing pathogens (8, 9, 10). Alternatively activated macrophage phenotype is mediated by a different set of cytokines such as IL-4, IL-10, and TGF-β. These macrophages promote tissue repair, wound healing, and resolution of inflammation (2, 3, 7, 10). However, recent studies employing single-cell RNA sequencing and spatial transcriptomics have uncovered the presence of numerous phenotypically and functionally distinct macrophage subsets that are driven by a range of environmental stimuli (7, 11, 12, 13, 14).

Epigenetic modifications and their cognate enzymes are being recognized as key contributing factors to the plasticity of macrophage phenotype and polarization (1, 15, 16, 17, 18). Histone deacetylation by HDAC3 and 9, histone methylation by SET7/9, DNA methylation by the DNA methyltransferases 1 and 3b (DNMT1, 3b), and DNA demethylation by Ten-eleven translocation 2 (TET2) have been shown to promote a pro-inflammatory macrophage phenotype through their enzymatic activities. The histone deacetylases HDAC4 and SIRT1 and the histone methyltransferases SMYD2-3 and PRMT1 have also been shown to facilitate an anti-inflammatory macrophage phenotype (1, 18). Mechanistically, upon stimulation with TLR ligands, open chromatin *loci* around inflammatory genes are primed by master transcription factors in quiescent macrophages (1, 19). In addition, repressive histone methylation marks in inflammatory gene *loci*, also termed “epigenetic brakes”, are removed by the lysine demethylases (KDMs) harboring the Jumanji C (JmjC) catalytic domain. KDMs belong to 2-oxoglutarate (2OG) and Fe^2+^-dependent dioxygenase superfamily, which also includes DNA and RNA demethylases TET2 and the fat mass and obesity associated protein FTO, respectively (20, 21). Several members of the KDM family, >30 in the human genome, play crucial roles in the regulation of immune cell development and differentiation through site-specific lysine demethylation in histones (22, 23, 24). Although these studies suggest that certain epigenetic modifiers are crucial to fuel macrophage-mediated inflammation, macrophage-specific epigenetic modifiers have not been targeted *in vivo* to modulate the function of this critical inflammatory cell subset.

To gain mechanistic insights and identify novel therapeutic targets, we screened a panel of both promiscuous and specific cell-permeable inhibitors against chromatin modifiers, including KDMs, and examined their effects on inflammatory pathways. Among all the examined inhibitors of chromatin modfiers, we show that the inhibition of the JmjC histone demethylases greatly diminishes the inflammatory response to LPS stimulation in macrophages *in vitro* and *in vivo*. Specifically, n-octyl-IOX1 (a *pan*-KDM inhibitor) (25) and PB-IT (a KDM5 inhibitor) (26) significantly decrease LPS-mediated inflammatory gene expression in murine bone marrow-derived macrophages (BMDM) by rewriting their epigenetic landscapes. Furthermore, selective delivery of n-octyl-IOX1 to macrophages using lipidoid nanoparticles (LNPs) severely dampened the inflammatory response to LPS in mice. Our study uncovers a crucial role of the JmjC-domain-containing KDMs in inflammatory macrophage polarization and demonstrates the therapeutic potential of JmjC inhibitors to suppress macrophage-mediated inflammation in disease.

## Results

### JmjC KDMs mediate pro-inflammatory response in macrophages

**Figure 1:**
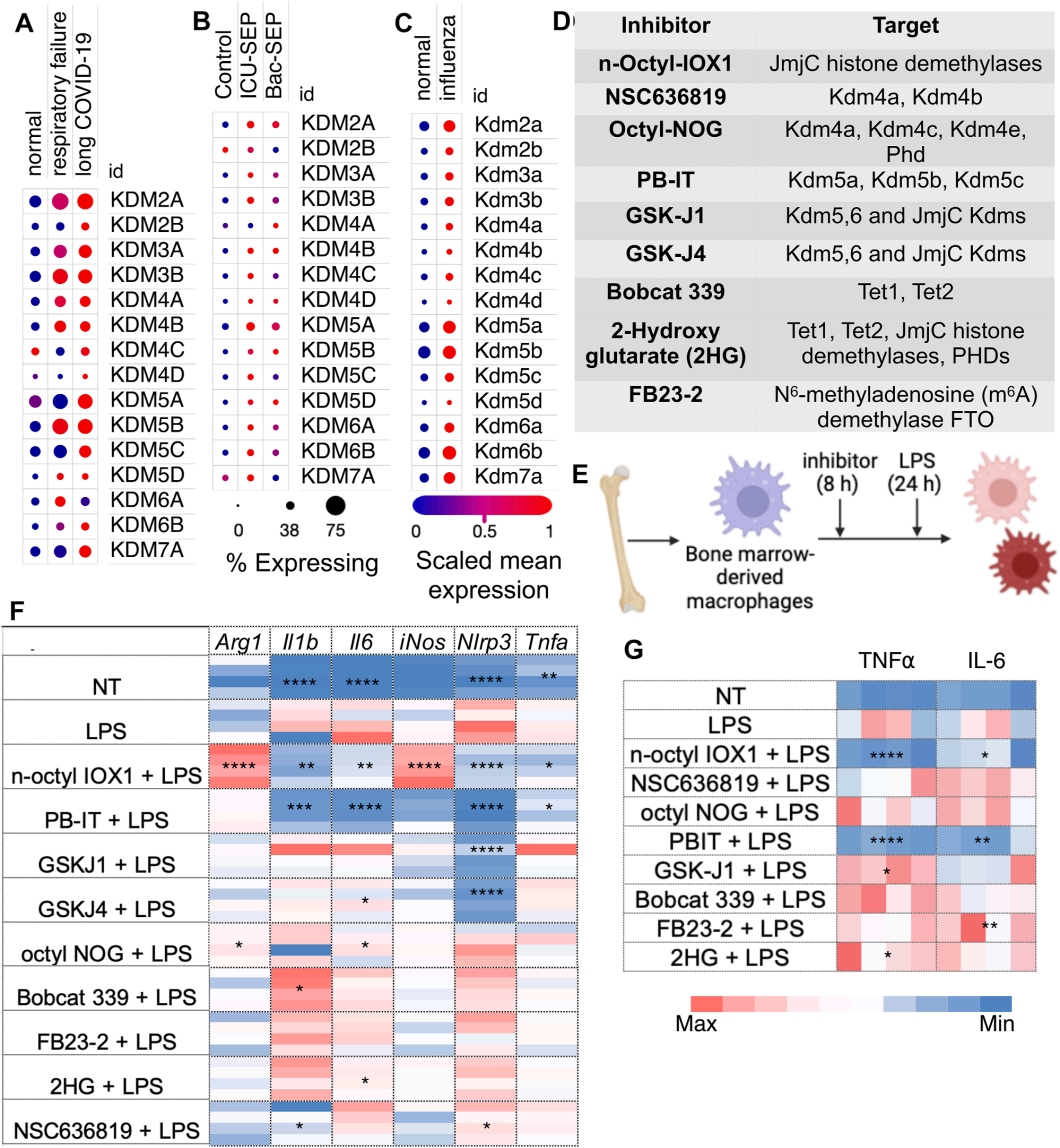
**JmjC histone demethylases regulate inflammatory macrophage polarization.** Expression of JmjC histone demethylase isoforms (KDMs 2, 3, 4, 5, 6, and 7) from Single Cell Portal (https://singlecell.broadinstitute.org/single_cell) in (*A*) lung macrophages of normal, respiratory failure, and long COVID-19 patients, (*B*) circulatory monocytes in control, ICU-sepsis, and bacterial sepsis patients, and (*C*) lung macrophages of normal and influenza-infected mice. *D*, the small molecule inhibitor panel employed in the study and target JmjC histone demethylases are summarized. *E*, the experimental design to screen small molecule JmjC inhibitors in bone marrow-derived macrophages is depicted. *F* and *G* Inflammatory gene expression was quantified by qPCR (*F*), and TNFA and IL-6 levels in supernatant were measured by ELISA (*G*). F,G: n = 4/group, ∗*p* < 0.05, ∗∗*p* < 0.01, ∗∗∗*p* < 0.001, ∗∗∗∗*p* < 0.0001, one-way ANOVA with *post hoc* Fisher LSD test. Significance shown in Figure 1, *F* and *G* is in comparison with the LPS-treated group.

The JmjC domain-containing KDMs have emerged as master regulators of eukaryotic gene expression and are known to play key roles in development, inflammatory disease, and malignancies. (22, 23). However, the role played by KDMs in macrophage polarization and inflammation in the context of disease is largely unexplored. To this end, we examined the expression profile of KDMs in three independent infectious disease contexts (human long COVID-19 (27), human bacterial sepsis (28), and mouse influenza (29)) using the open-source data at Broad Institute’s Single cell database (https://singlecell.broadinstitute.org/single_cell). Alveolar macrophages of patients with respiratory failure and long-COVID-19 expressed higher levels of KDMs, such as KDM2A, KDM3A-B, KDM4A-B, KDM5B, and KDM5D, compared to healthy individuals (Fig. 1*A*) (27). In line with this observation, circulating monocytes of patients with intensive care and bacterial sepsis exhibited elevated expression of various KDMs compared to the control cohort (Fig. 1*B*) (28). Furthermore, murine lung macrophages had elevated expression of the KDMs after exposure to the influenza virus (Fig. 1*C*) (29).

To characterize the function of KDM enzymes in macrophage polarization and inflammation, we performed a chemical screen wherein murine BMDMs were pre-treated with small-molecule inhibitors of KDMs prior to LPS stimulation (Table 1), and the genes known to be expressed by pro- and anti-inflamamtory macrophages were evaluated (Fig. 1, *D* and *E*,). Pre-treatment of macrophages with the *pan*-KDM inhibitor n-octyl-IOX1, significantly decreased the expression of the inflammatory cytokines *Il1b*, *Il6*, and *Tnfa* and the *Nlrp3* inflammasome (Fig. 1*F*). n-Octyl-IOX1 also increased *Arg1*, which is a marker of anti-inflammatory and pro-resolving macrophages. The KDM5 inhibitor PB-IT also lowered the expression of the inflammatory genes *Il1b*, *Il6*, and *Nlrp3*. On the contrary, the inhibitors against KDM4 (n-octyl-NOG and NSC636819), KDM5,6, and JmjC KDMs (GSK-J1 and GSK-J4 (30), TET1 and TET2 (Bobcat 339 and 2HG), and FTO (FB23-2) were not as efficient as n-octyl-IOX1 and PB-IT in suppressing the expression of the pro-inflammatory genes. Consistent with the diminution of *Tnfa* and *Il6* genes, the treatment of n-octyl-IOX1 and PB-IT, but not the other inhibitors, significantly curtailed TNF-⍺ and IL-6 production by macrophages in response to LPS (Fig. 1*G*). We further noted that the expression of *Mmp14*, which encodes an enzyme implicated in extracellular matrix assembly, was downregulated in n-octyl-IOX1 and PB-IT-treated macrophages (Fig. S1). Furthermore, these treatments significantly lowered the expression of *Ccl5* and *Ccl7*, which are shown to orchestrate inflammatory macrophage polarization (31). Therefore, we demonstrate that n-octyl-IOX1 and PB-IT significantly dampen the inflammatory response to LPS in macrophages.

**Table 1:** The KDM inhibitors used in this study are listed with their sources and concentrations used

Inhibitor	Vendor, catalog#	Concentration
n-Octyl-IOX1	Selleckchem, S7234	20 mM
PB-IT	Selleckchem, E1733	20 mM
GSK-J1	Selleckchem, S7581	1 mM
GSK-J4	Selleckchem, S7070	1 mM
Octyl-NOG	Cayman chemical, 5262-39-5	25 mM
Bobcat 339	Selleckchem, S6682	2 mM
FB23-2	Selleckchem, S8837	1 mM
2-Hydroxy glutarate (2HG)	Selleckchem, S7873	25 mM
NSC636819	Medchemexpress HY-110154	25 mM

### KDM inhibitors suppress transcriptome-wide inflammatory pathways in LPS-stimulated macrophages

**Figure 2:**
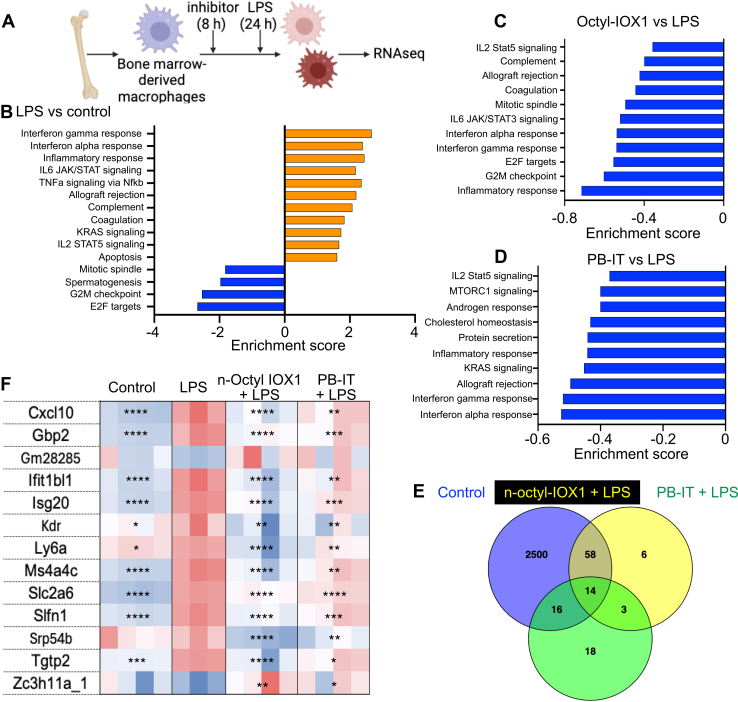
**LPS-mediated inflammatory pathways in macrophages are downregulated by the JmjC inhibitors n-octyl-IOX1 and PB-IT.***A*, experimental strategy: RNAseq was performed in murine BMDM pre-treated with n-octyl-IOX1or PB-IT for 8 h followed by LPS stimulation for 24 h. *B–E*, the pathway analyses show the significantly altered pathways (*p* < 0.05) between LPS vs. control (*B*), n-octyl-IOX1 vs. LPS (*C*), and PB-IT vs. LPS (*D*). *E*, the Venn diagram unveils the overlaps of the differentially expressed genes in control, n-octyl-IOX1 + LPS, and PB-IT + LPS compared to the LPS group, fold change >1.5, FDR *p* < 0.05. *F*, heatmap showing the expression of the common 14 genes differentially expressed in the groups shown in E. *G*, Wordcloud representation of GeneAgent analysis of conserved genes identified in F. Fold change >1.5, FDR *p* < 0.05. n = 3 (LPS), 4 (all other groups). ∗*p* < 0.05, ∗∗*p* < 0.01, ∗∗∗*p* < 0.001, ∗∗∗∗*p* < 0.0001, one-way ANOVA with *post hoc* Fisher LSD test.

As discussed earlier, among all examined inhibitors, n-octyl-IOX1 and PB-IT efficiently restricted the LPS-elicited inflammatory response in macrophages. This finding prompted us to perform a transcriptome-wide gene expression analysis in BMDM treated with the inhibitors prior to stimulation with LPS (Fig. 2*A*). As anticipated, pathway analyses clearly showed that LPS treatment upregulated several inflammatory networks in macrophages (Figs. 2*B* and S2). Interferon gamma response, interferon alpha response, IL-6 - JAK/STAT signaling, TNF-⍺ signaling, complement, and IL-2 Stat5 signaling were enriched in LPS-treated cells compared to vehicle treatment. In concordance with our targeted gene expression and ELISA data, pre-treatment with n-octyl-IOX1 or PB-IT significantly dampened the transcriptome-wide inflammatory response to LPS in macrophages (Figs. 2, *C* and *D*, S3, and S4). n-Octyl-IOX1 treatment downregulated the IL2-STAT5, complement, coagulation, IL6 JAK/STAT3 signaling, interferon alpha response, and interferon gamma response pathways. PB-IT inhibited IL2 STAT5, MTORC1 signaling, and KRAS signaling. The common pathways downregulated by n-octyl-IOX1 and PB-IT include IL2-STAT5 signaling, allograft rejection, inflammatory response pathway, interferon gamma response, and interferon alpha response (Fig. 2, *C* and *D*). We identified 14 common differentially expressed genes after the treatments compared to LPS stimulation, as depicted in the Venn diagram (Fig. 2*E*). The expression of these genes is depicted in the heatmap (Fig. 2*F*). Notable examples include the inflammatory chemokine *Cxcl10*, the interferon response gene *Ifit1bl1* and the regulator of glycolysis *Slc2a6*. Cxcl10, a chemoattractant, recruits immune cells to sites of injury or inflammation and mediates a pro-inflammatory response selectively in macrophages (32). Taken together, these data indicate that n-octyl-IOX1 and PBIT decrease chemokine production and rewire interferon response genes in macrophages.

### n-Octyl-IOX1 rewires LPS-induced epigenetic alterations in macrophages

**Figure 3:**
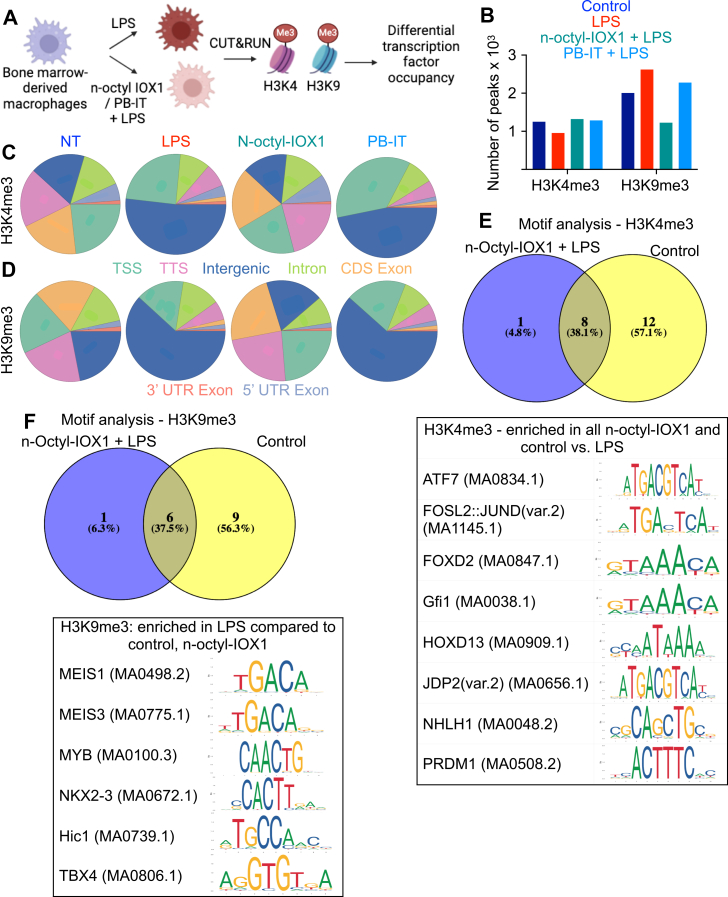
**n-octyl-IOX1 reverses the epigenetic changes induced by LPS in macrophages.***A,* CUT&RUN for H3K4me3 and H3K9me3 was performed on murine BMDM pretreated with n-octyl-IOX1, PB-IT, or GSK-J1 for 8 hours followed by LPS stimulation for 24 hours. *B–F,* CUT&RUN data analyses were performed to assess the (*B*) number of peaks and (*C* and *D*) distribution of identified peaks at transcription start site (TSS), transcription termination site (TTS), intergenic, intron, CDS exon, 3’ and 5’ UTR exon (*C*: H3K4me3, *D*: H3K9me3). *E* and *F*, the Venn diagram represents differentially enriched motifs for H3K4me3 (*E*) and H3K9me3 (*F*). The motifs enriched in all conditions *versus* LPS treatment are summarized in the tables below the corresponding Venn diagrams. LPS, lipopolysaccharide.

We next sought to investigate if n-octyl-IOX1 and PB-IT prevent LPS-induced epigenetic alterations in macrophages. Since n-octyl-IOX1 is a pan-JmjC KDM inhibitor, we hypothesize that it preserves the naive-state histone methylation landscape in LPS-responsive genes in macrophages. As a complex network of activating and repressive marks regulate LPS-induced inflammatory responses in macrophages, we postulated that n-octyl-IOX1 likely rewires both activating and repressive marks to dampen inflammatory responses to LPS. To this end, we performed cleavage under targets and release using nuclease (CUT&RUN) assay followed by next-generation sequencing to examine the changes in H3K4me3 (an active histone mark) and H3K9me3 (a repressive histone mark) in BMDM (Fig. 3*A*) as n-octyl-IOX1 is an inhibitor of all JmjC histone demethylases. Overall, LPS treatment altered the number of H3K4me3 peaks and increased H3K9me3 peaks (Fig. 3*B*). The transcription startsite plots of CUT&RUN peaks show that the distribution of most of the peaks was around the transcription start sites in LPS-exposed macrophages (Fig. S5). However, n-octyl-IOX1, but not PB-IT, treatment reduced LPS-mediated peak distribution and exhibited a peak distribution similar to the control group (No treatment) (Fig. 3, *B*–*D*). Congruently, the differentially enriched regions contained eight overlapping motifs (38.1%) for the H3K4me3 mark enriched in the n-octyl-IOX1 and control groups, compared to LPS (Figs. 3*E* and S6*A*). The eight common motifs enriched in n-Octyl-IOX1 and control include FOSL2:JUND, PRDM1, and NHLH1, which are known to be pivotal for suppressing macrophage-mediated inflammation. FOSL2 is a transcriptional regulator of M2-like pro-reparative macrophage polarization (33), and PRDM1 (*Blimp1*) is a key transcriptional repressor of chemokine genes and is required for macrophage differentiation (34). The results suggest that n-octyl-IOX1 treatment exerts epigenetic marks similar to the control group.

We identified six common H3K9me3 motifs to be enriched in the n-octyl-IOX1 and control groups compared to the LPS-treated group (Fig. 3*F*). The genes containing these motifs are MEIS1, MEIS3, MYB, NKX2.3, Hic1, and TBX4 (Figs. 3*F* and S6*B*). NKX2.3 is a transcription factor that regulates hematopoiesis and lymphopoiesis, and the loss of NKX2.3 disrupts macrophage architecture in the splenic marginal zone (35). In aggregate, these findings suggest that n-octyl-IOX1 reverses the epigenetic alterations provoked by LPS in macrophages.

### n-Octyl-IOX1 dampens inflammatory response to LPS *in vivo*

**Figure 4:**
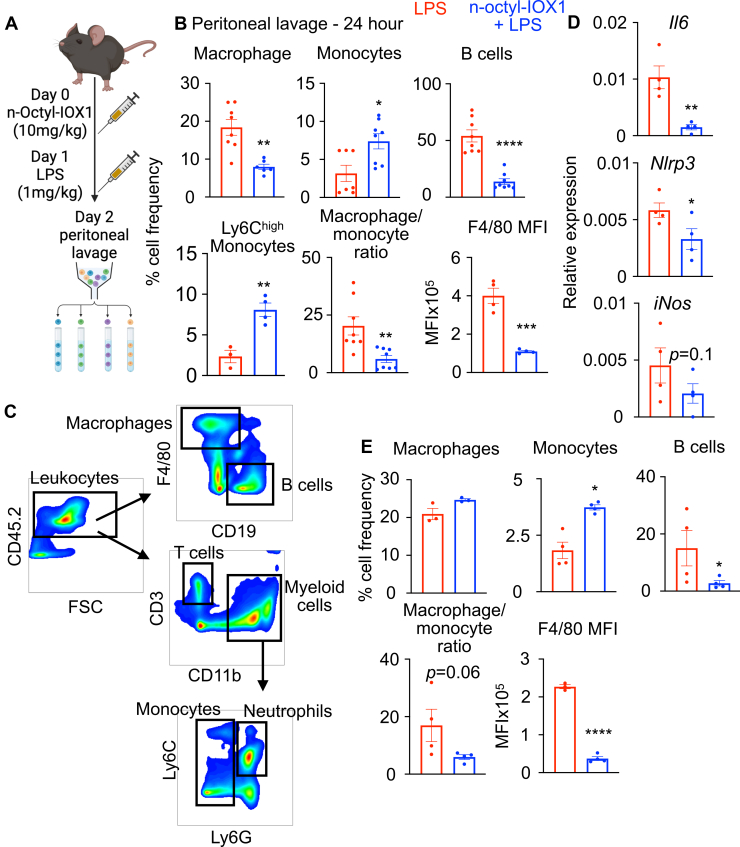
**n-octyl-IOX1 lowers inflammatory gene expression *in vivo*.***A*, experimental design: n-octyl-IOX1 (10 mg/kg) was administered into C57BL/6 mice intraperitoneally, followed by 1 mg/kg LPS after 24 h. *B*, peritoneal lavage was collected 24 h or 48 h after LPS administration and subjected to flow cytometry. The frequencies of leukocyte subsets in peritoneal lavage 24 h after LPS injection were calculated. *C*, the gating strategies to sort macrophages, *B* cells, monocytes, and neutrophils from peritoneal lavage are provided. *D*, the expression of *Il6*, *Nlrp3*, and *iNos* in peritoneal macrophages 24 h after LPS was calculated. *E*, the frequencies of leukocyte subsets in peritoneal lavage 48 h after LPS and n-octy-IOX1 were quantified by flow cytometry. ∗*p* < 0.05, ∗∗*p* < 0.01, ∗∗∗*p* < 0.001, ∗∗∗∗*p* < 0.0001, 2-tailed Student’s *t* test. n = 4–6/group.

Our data demonstrate that the histone demethylase inhibitors, especially n-octyl-IOX1, effectively diminished macrophage-mediated inflammation, which is key to the progression of several diseases. We also demonstrated downregulation of inflammatory pathways and a rewiring of H3K4me3 and H3K9me3 motifs upon n-octyl-IOX1 treatment in primary macrophages. Next, we sought to examine the therapeutic potential of n-octyl-IOX1 *in vivo*. To this end, we examined the effect of n-octyl-IOX1 on dampening macrophage-mediated inflammatory response to LPS *in vivo*. Ten-week-old female C57BL/6 mice were pre-treated with n-octyl-IOX1, followed by LPS priming (Fig. 4*A*). We enumerated leukocytes by flow cytometry 24 h post-LPS administration. n-octyl-IOX1 treatment decreased the frequency of macrophages and increased monocyte proportions in peritoneal lavage (Fig. 4*B*). We observed a reduction in total monocyte and Ly-6C^high^ monocyte frequencies in the peripheral blood of the inhibitor-treated group compared to the vehicle-treated group at 24 h after treatment (Fig. S7). However, interestingly, this reduction in monocyte and Ly-6C^high^ monocyte frequencies was lost at 48 h. We observed diminished ratios of peritoneal macrophages to monocytes, suggesting impaired monocyte to macrophage differentiation and/or higher Ly-6C^high^ monocyte influx into the peritoneal cavity after n-octyl-IOX1 treatment. The frequency of B cells was lower in the peritoneal lavage, while Ly-6C^high^ monocyte frequency was elevated (Fig. 4*B*). The expression of the inflammatory genes *Il6* and *Nlrp3* was significantly dampened in sorted peritoneal macrophages upon n-octyl-IOX1 administration (Fig. 4, *C* and *D*). We also observed a similar reduction in macrophage/monocyte ratios, B cells, and F4/80 MFI at 48 h after n-octyl-IOX1 treatment (Fig. 4*E*). In summary, these data indicate that a global, non-targeted administration of n-octyl-IOX1 restrained inflammatory respnse resulting from *in vivo* LPS stimulation.

### Macrophage-specific delivery of n-octyl-IOX1 limits inflammatory gene expression *in vivo*

**Figure 5:**
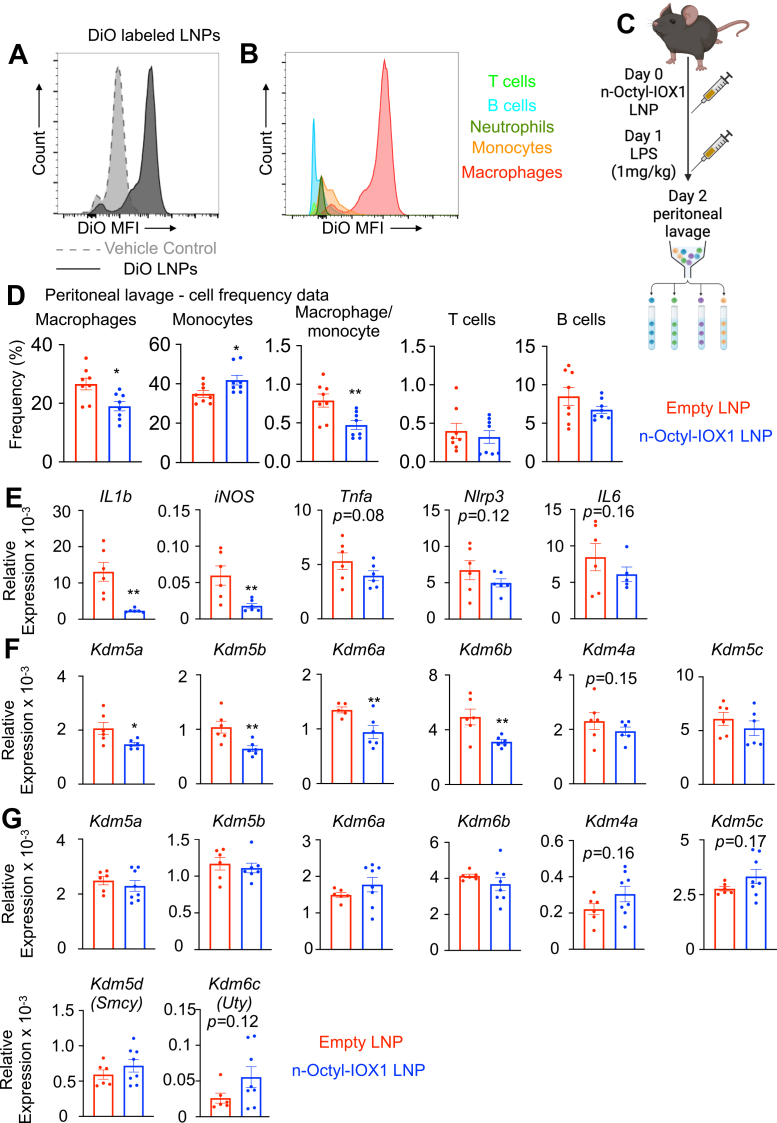
**Macrophage-specific n-octyl-IOX1 delivery using lipidoid nanoparticles suppresses inflammation.***A*, MFI of DiO in peritoneal macrophages of mice after i.p administration of DiO-n-octyl IOX1 lipidoid nanoparticles is shown. *B*, DiO MFI is depicted in various cell types. *C*, the study design to examine the effects of macrophage-specific n-octyl-IOX1 delivery is depicted. *D*, Flow cytometry was performed to evaluate the leukocyte frequencies in peritoneal lavage of the mice administered with empty LNP or n-octyl-IOX1 LNP. *E* and *F*, the expression of the inflammatory genes (*E*) and the KDM isoforms in in sorted peritoneal macrophages of female (*F*) and male (*G*) C57BL/6 animals was discerned. ∗*p* < 0.05, ∗∗*p* < 0.01, 2-tailed Student’s *t* test. n = 8/group (*D*), 6 to 8/group (*E–G*).

Our results show that KDMs promote pro-inflammatory macrophage polarization and n-octyl-IOX1 impedes the inflammatory response to LPS *in vitro* and *in vivo*. To avoid off-target effects of n-octyl-IOX1 due to non-targeted administration, we sought to deliver the inhibitor specifically to macrophages using lipidoid nanoparticles (LNPs). Although LNPs have been used to successfully carry out siRNA-mediated gene silencing, whether the same strategy can be adopted to deliver small molecule inhibitors against epigenetic modifiers into macrophages *in vivo* is not known. To this end, we employed DiO-labeled n-octyl-IOX1-loaded DOPC/DSPG LNPs (DOPC: 1,2-dioleoyl-sn-glycero-3-phosphocholine, DSPG: 1,2-distearoyl-sn-glycero-3-phospho-rac-1-glycerol, Fig. S8, *A* and *B*, size distribution of LNPs). We confirmed macrophage-specific uptake of empty and n-octyl-IOX1-loaded LNPs in C57BL/6 mice by flow cytometry (Fig. 5, *A* and *B*). Next, 10-week-old female C57BL/6 mice were treated with either n-octyl-IOX1 LNP or empty LNP, followed by LPS-mediated immune activation (Fig. 5*C*). n-Octyl-IOX1 LNP treated animals exhibited a significant reduction in peritoneal macrophage frequency and macrophage/monocyte ratio, suggesting suppressed monocyte to macrophage differentiation (Fig. 5*D*). However, T cell frequencies were elevated. We also assayed leukocyte frequencies in the blood, bone marrow, and spleen of these mice by flow cytometry (Fig. S8, *C* and *E*). Neutrophil frequency was suppressed in the blood and bone marrow of octyl-IOX1 cohort. Moreover, T cells were decreased in the bone marrow. Macrophage/monocyte ratios were elevated in the bone marrow while these ratios were reduced in the spleen, suggesting higher retention of macrophages, which are pathogenic, in the bone marrow. Next, to assay inflammatory gene expression, we sorted peritoneal macrophages of mice treated with either empty or n-octyl-IOX1 LNPs. *Il1b*, which has been shown to be crucial in inflammatory disease progression (36, 37), and *iNos*, which is a driver of inflammatory macrophages (38, 39), were both downregulated in the macrophages of n-octyl-IOX1 LNP-treated mice while there was a non-significant decrease in *Tnfa*, a key driver of inflammation (Fig. 5*E*). Interestingly, we also observed a reduction in the expression of several JmjC KDMs in peritoneal macrophages of n-octyl-IOX1 LNP-treated female animals compared to empty LNP controls (Fig. 5*F*). Interestingly, peritoneal macrophages from n-octyl-IOX1 treated male animals did not exhibit a similar reduction in JmjC KDMs (Fig. 5*G*). We did not observe downregulation of inflammatory gene expression in sorted peritoneal monocytes, neutrophils or B cells, further confirming cell-targeted effects of n-octyl-IOX1 LNPs (Fig. S9, *A* and *C*). Some members of the KDM-family are encoded on the X chromosome, such as KDM5C (SMCX) and KDM6A (UTX) or the Y chromosomes, such as KDM5D (SMCY) and KDM6C (UTY). To examine the effects of JmjC KDM inhibition on KDM5D and KDM6C, we administered empty or n-octyl-IOX1 LNPs to 10-week-old female and male C57BL/6 mice followed by LPS stimulation (Fig. 5*A*). Interestingly, we observed a reduction in Ly-6C^high^ monocyte numbers in peritoneal lavage and increased macrophage/monocyte ratios in male mice (Fig. S8*F*). While *Kdm5b* and *Kdm6a* were decreased by n-octyl-IOX1 LNP administration in peritoneal macrophages isolated from female mice (Fig. 5*F*), we did not observe significant alterations in the expression of Kdm5c in female peritoneal macrophages (Fig. 5*F*) or *Kdm5d* and *Kdm6c* in male peritoneal macrophages (Fig. 5*G*).

Collectively, our data demonstrate that macrophage-specific epigenetic modifiers, such as the KDMs, can be targeted *in vivo* to alleviate inflammation.

## Discussion

Innate immune memory, wherein innate immune cells retain cellular “memory” of prior pathogens and exhibit an amplified immune response upon restimulation, is an emerging area of study with an immense significance in immunology (40, 41). Innate memory and resultant trained immunity are primarily mediated by epigenetic imprinting mechanisms (42). Observed in various myeloid cell populations, trained immunity persists for prolonged periods of time due to epigenetic alterations of hematopoietic and myeloid progenitor cells in the bone marrow (43). Increased risk of subsequent infections and mortality following infections or sepsis is attributed to long lasting aberrant epigenetic reprogramming of myeloid cells (44). Furthermore, myocardial infarction mediates long-term reprogramming of monocytes to a pro-inflammatory state *via* epigenetic alterations accelerating atherogenesis and inflammation (43, 45, 46).

Aberrant histone demethylation disrupting gene regulatory mechanisms is attributed to the pathogenesis of inflammatory disorders resulting in organ damage (47). Owing to the ability to demethylate both activating (*e.g.*, H3K4me3 and H3K36me3) and silencing (*e.g.*, H3K9me3 and H3K27me3) histone marks, KDMs have shown to play essential roles in inflammatory responses in diverse immune cell types (22, 23). There is a large body of research supporting the role of KDMs in activation of both innate and adaptive immune cells (47, 48). In macrophages, KDM6A enhances IL-6 and IL-1β secretion by demethylation of the repressive H3K27me3 mark in the promoter region (49). KDM6B is known to regulate inflammatory gene expression in macrophages in response to LPS and in diabetic wound healing (50, 51). The H3K4me3 demethylase KDM5B recruits H3K9 methyltransferase SETDB1 to suppress anti-tumor immunity (52). Treatment with oxidized low-density lipoprotein (oxLDL) upregulates KDM4A expression in macrophages, and silencing KDM4A reverses the inflammatory response to oxLDL (53). Our analyses of open-source single-cell transcriptomic data show that KDM isoforms are upregulated in murine and human macrophages after infection (Fig. 1). These data are congruent with the reports that dysregulated expression of KDMs is implicated in the pathogenesis of infectious diseases (47, 54). Furthermore, KDMs have been implicated in autoimmune disorders, such as inflammatory bowel disease, and tumor progression (48, 55, 56, 57). Due to the crucial roles played by KDMs in various aspects of pathophysiology, several studies have focused on the development of small-molecule antagonists, which need to be validated in clinical trials (58, 59, 60). Therefore, small-molecule inhibitors of KDMs are critical to investigate the function of KDMs in cell types of interest and develop novel therapeutics targeting inflammation in infection, autoimmunity, and sepsis (55, 57).

In this study, we evaluated small-molecule inhibitors of KDMs to suppress inflammatory responses in macrophages *in vitro* and *in vivo*. Pre-treatment with n-octyl-IOX1 and PB-IT restrained the inflammatory response to LPS in primary macrophages. The *pan*-KDM inhibitor n-octyl-IOX1 downregulated inflammatory pathways and rewired gene regulatory pathways, restoring key inflammatory gene expression signature and epigenetic state of macrophages similar to that of the control state. Furthermore, although the macrophage epigenetic landscape can be altered *in vitro* using small-molecule inhibitors against chromatin modifiers, the delivery of these inhibitors specifically to macrophages *in vivo* to avoid any off-target effects has been challenging. We employed a novel LNP-mediated macrophage-specific delivery of n-octyl-IOX1 *in vivo* and demonstrated that this strategy decreased inflammatory macrophage numbers and mitigated the production of inflammatory cytokines in mice injected with LPS. Since the mechanisms of action of the JmjC KDMs in various cell types in the context of disease *in vivo* remain to be fully understood, such targeted delivery of KDM agonists/antagonists holds immense therapeutic potential with reduced off-target effects and adverse drug reactions (61, 62). Interestingly, we also observed a reduction in the expression of Kdm5 and Kdm6 isoforms upon treatment with n-octyl-IOX1, indicating the presence of a feedback loop wherein the inhibitor downregulates both the expression of JmjC Kdms. Furthermore, we observed that macrophage-specific n-octyl-IOX1-LNP delivery elicited a targeted immunomodulatory effect, while *IL1b* and *iNos* were significantly reduced, *Nlrp3* inflammasome and *Il6* levels were decreased marginally, compared to systemic n-octyl-IOX1 administration. We postulate that this is due to complex paracrine signaling among immune cell subsets that shape inflammatory responses to LPS *in vivo*.

A notable observation from our CUT&RUN analyses is that n-octyl-IOX1 rewires both activating and repressive histone methylation marks, conventionally attributed to opposing transcriptomic programs. We hypothesize that this is an outcome of pan-JmjC KDM inhibition, as the JmjC family contains KDMs that target both activating and repressive histone marks (23). LPS-responsive induction of inflammatory programs in macrophages requires the coordinated removal of inhibitory marks to counter baseline homeostatic repression, as well as the accumulation of activating methylation marks to promote transcription of target genes (1, 63, 64). Studies have shown that inhibitors of individual KDMs affect either activating or repressive marks: GSK-J4 affects H3K27me3 levels (30, 57), while PB-IT modulates H3K4me3 demethylation (26). However, neither GSK-J1 nor PB-IT demonstrated the transcriptomic and epigenetic reprogramming of LPS-stimulated macrophages achieved by n-octyl-IOX1 through the parallel rewiring of activating and repressive marks, as demonstrated by our CUT&RUN experiment.

While we demonstrate the immunomodulatory role of the JmjC KDMs and the therapeutic potential of macrophage-specific delivery of n-octyl-IOX1 as a proof-of-concept application, several limitations must be addressed. Firstly, n-octyl-IOX1 competitively inhibits members of the 2OG-dependent dioxygenases, including DNA and RNA demethylases, and prolyl hydroxylases (PHDs) in addition to KDMs. This makes it challenging to isolate the KDM-sepcific mechanism of n-octyl-IOX1 in macrophages. However, small molecule inhibitors specific for other members of the JmjC KDM family have been developed, including NSC636819 (KDM4A), PB-IT (KDM5B), and GSK-J1/J4 (KDM6A) (65). Our initial screening of these inhibitors indeed demonstrated the ability of PB-IT to silence inflammatory genes in BMDMs. We observed varied function of the different inhibitors in our study, highlighting the functional diversity of the JmjC KDMs. Furthermore, we observed a sex-dependent effect of n-octyl-IOX1 on macrophage inflammation *in vivo*. Macrophage/monocyte ratios were increased in male animals, while it was decreased in female animals upon n-octyl-IOX1-LNP administration. This observation, combined with the reduction in *Kdm6a* (*Utx*) in female peritoneal macrophages, but not *Kdm5d* (*Smcy*) and *Kdm6c* (*Uty*), which are encoded by the Y chromosome, in male peritoneal macrophages, points toward sex-dependent regulation of KDM-mediated macrophage function. We observed a reduction in total monocyte and Ly-6C^high^ monocyte frequencies in the peripheral blood of the inhibitor-treated group compared to the vehicle-treated group at 24 h after treatment (Fig. S7). However, interestingly, this reduction in monocyte and Ly-6C^high^ monocyte frequencies was lost at 48 h, which could be due to compensatory monocyte production by the bone marrow at the latter time point. Future studies will employ these small-molecule inhibitors to evaluate the member-specific role of KDMs in the context of macrophage-mediated inflammation *in vivo*. Given that we assessed inflammatory gene expression in the murine model of peritonitis with non-lethal LPS doses, further investigations will be required to elucidate the function of specific KDM in the context of sepsis and inflammatory diseases. Finally, histone demethylases also regulate higher order regulatory structures beyond discrete structural motifs. These higher order motifs composed of composite elements or fuzzy and continuous motifs regulated by KDMs may modulate inflammatory gene expression in macrophages (66, 67). Additional studies will be required to delineate the role KDMs in modulating higher order regulatory structures in macrophages.

## Experimental procedure

### Animals

All animal experiments were performed according to the NIH guidelines, and the protocols of the animal experiments were approved by the University of Pittsburgh Institutional Animal Care and Use Committee. Adult C57BL/6 mice (#000664) were obtained from the Jackson laboratory. All animals were maintained in a standard 12-h light/dark cycle in the vivarium. 10-to twelve-week-old mice were used for experiments. Bone marrow was collected for flow cytometry or BMDM culture. Peritoneal lavage, spleen and blood were collected for flow cytometry and cell sorting. 10 mg/kg n-octyl-IOX1 and macrophage-targeted n-octyl-IOX1 incorporated in lipidoid nanoparticles, and 1 mg/kg LPS were administered intraperitoneally. Vehicle or empty lipidoid nanoparticle-treated groups were used as controls. Twenty-four hours after systemic 10 mg/kg n-octyl-IOX1 or n-octyl-IOX1 lipidoid nanoparticle administration, 1 mg/kg LPS was administered intraperitoneally. Leukocyte subsets in peritoneal lavage, blood, bone marrow, and spleen were assayed 24 and 48 h post-LPS administration by flow cytometry. Macrophages, monocytes, neutrophils, and B cells were sorted from peritoneal lavage to assess inflammatory gene and histone demethylase expression.

### BMDM culture, inhibitor, and LPS treatment

Bone marrow cells were isolated from the femurs and tibias of C57BL/6 mice. The cells were cultured in DMEM containing 5.5 mM glucose and supplemented with 20% L929 conditioned medium. The cells were seeded in 12-well plates. After the cells were 80% confluent, the culture was treated with epigenetic inhibitors for 8 h, followed by 1 ng/ml LPS for 24 h prior to the collection of cells for qPCR and conditioned medium for ELISA.

### ELISA

IL6, IL1B, and TNFA were measured in conditioned cell culture medium according to manufacturer’s instructions. Briefly, 96-well plates were coated with capture antibody. Samples were incubated in coated plates overnight, followed by incubation with detection antibody, and HRP-conjugated antibody. TMB substrate solution and 1N sulfuric acid were added, followed by measurement of absorbance at 450 nm.

### Quantitative polymerase chain reaction

Qiagen RNeasy RNA isolation kit was used to extract total mRNA. cDNA was synthesized from 50 ng mRNA using the high-capacity RNA to cDNA synthesis kit (Applied Biosystems). SYBR green mastermix (Applied biosystems) was used to perform qPCR on a Quantstudio five system. Relative expression was estimated from the normalized C_t_ values.

### n-Octyl-IOX1 formulation in lipidoid nanoparticles

n-ocytl-IOX1 was formulated in phosphatidyl serine nanoparticles by Creative Biolabs. N-octyl-IOX1 was encapsulated at 30 mM in the LNPs. The LNPs were composed of DOPC/DSPG (90/10, mol/mol) with DiO at 0.01 mol%, in a buffer of 300 mM sucrose with 0.5% DPBS (1X). Size distribution of empty and n-octyl-IOX1 LNPs are shown in Figure. S8, *A* and *B*. Selective uptake of LNPs was assessed by flow cytometry in peritoneal lavage.

### Organ harvesting and flow cytometry

Mice were euthanized according to the University of Pittsburgh IACUC guidelines. Blood was collected by terminal cardiac puncture and incubated with RBC lysis buffer for 3 min at room temperature, followed by addition of FACS buffer and centrifugation to pellet leukocytes. A hemocytometer was used to count the number of viable cells in the organs.

The following panel of antibodies were used to analyze myeloid cell populations in mice: CD45.2 (Biolegend, #109,820, clone 104, lot B364148), CD11b (BD Biosciences, #557,657, clone M1/70, lot 1271559), CD115 (Biolegend, #565,249, clone T38-320, lot 7291701), Ly6G (Biolegend, #563,979, clone 1A8, lot 0269936), Ly-6C (eBioscience, #45-5932-82, clone HK1.4, lot 2162018), F4/80 (Biolegend, #123114, clone BM8, lot B370041) CD3 (BD Biosciences, #367-0032-82, clone 17A2, lot 2526889), and CD19 (BD Biosciences, #563148, clone 1D3, lot 3130912). Neutrophils were identified as CD11b^+^ Ly6G^+^ and CD115^−^. Monocytes were considered as CD11b^+^ Ly-6G^-^ and CD115^+^. B and T Lymphocytes were identified as CD11b^-^ CD19^+^ CD3^-^ and CD11b^-^ CD19^-^ CD3^+,^ respectively. Peritoneal macrophages were determined as CD45.2^+^ CD11b^+^ Ly6G^−^ F4/80^+^ and sorted into Picopure (Thermo Scientific) extraction buffer for qPCR. A Cytek Aurora flow cytometer was used to acquire the data, which were analyzed with the FlowJo software (Tree Star).

### RNA isolation and sequencing

RNA was isolated according to the manufacturer’s protocol (Qiagen RNeasy RNA isolation kit, #74106). Library prep and RNA sequencing was performed by the Health Science Sequencing core at UPMC Children’s hospital, Pittsburgh. RNA quality was assessed by an Agilent TapeStation 2200 using the High Sensitivity RNA Tape (Agilent: 5567–5579). Samples (5.5 μl) were used for cDNA generation using the Takara Smart-Seq HT kit (Takara, #634438) with 15 cycles of cDNA amplification following manufactures instructions. Smart-Seq cDNA was assessed for quality on an Agilent TapeStation 2200 using the D5000 High sensitivity ScreenTape (Agilent, #5067–5592). All samples passed QC with full length cDNA were advanced to library preparation using Illumina Nextera XT kit (Illumina: FC-131-1096) with 12 PCR cycles. Libraries were pooled and sequenced on an Illumina NextSeq 500 using a High Output 150 v2.5 cycle flow cell (Illumina: 20,024,907), 2 × 75 bp, for an average of ∼40 million reads per sample.

### RNA-sequencing data analysis

CLC genomics workbench was used to analyze transcriptomic data generated by RNA sequencing. The data generated from our RNAseq experiments have been deposited to GEO (accession numbers GSE292494 and GSE292495) and will be publicly released after the official acceptance of this manuscript. The FASTQ files provided by Health Science Sequencing Core at UPMC Children’s Hospital were imported into CLC Genomics Workbench 22. A sequencing QC report was generated to assess data quality. A PHRED score of less than 40 is considered optimal for downstream analysis. The adapters present at the ends were trimmed.

Forward - AGATCGGAAGAGCACACGTCTGAACTCCAGTCA.

Reverse - AGATCGGAAGAGCGTCGTGTAGGGAAAGAGTGT.

The trimmed reads were aligned to the *Mus musculus* CLC reference genome version 86. The gene expression was calculated as reads per kilobase of transcript per million mapped reads (RPKM). Next, differential expression of genes in pairwise comparisons was calculated. Differentially expressed genes were identified with at least 2 log2 fold change and FDR-corrected *p* value < 0.05. Venn diagrams were created using Venny 2.1 (Oliveros, J.C. (2007–2015) Venny. An interactive tool for comparing lists with Venn's diagrams. https://bioinfogp.cnb.csic.es/tools/venny/index.html).

### Pathway analysis

Pathway analyses were performed on the differentially expressed gene sets from RNAseq data using WEBGESTALT (68).

### CUT&RUN

CUT&RUN was performed according to manufacturer’s suggested protocol (Cell Signaling, #86652). Briefly, 100,000 BMDMs were plated per well, treated with epigenetic inhibitors and LPS as described above. Cells were trypsinized and harvested after treatment, permeabilized, and incubated with H3K4me3 and H3K9me3 antibodies overnight. Next, protein A-MNase was added to the cells after washing and incubated for 1 h. Then, chromatin digestion was performed, followed by release of digested chromatin and purification of digested DNA. Library preparation of CUT&RUN generated fragments was carried out, followed by sequencing (∼5million reads/sample).

### CUT&RUN data analysis

CUT&RUN data analysis was performed using Partek Flow. Sequences were aligned to the mouse genome, and adapters were trimmed followed by peak calling using MACS and peak annotation. From the generated peaks, known motifs and *de novo* motifs were identified. Gene set enrichment and pathway analyses were performed with the annotated peak data.

### Statistical analysis

Data are displayed as mean ± SEM. Statistical significance between groups was performed using Graphpad PRISM with Mann-Whitney, Student’s *t* test or ANOVA according to the dataset. Results were considered as statistically significant when *p* < 0.05.

## Data availability

The data generated from our RNAseq experiments have been deposited to GEO (accession numbers GSE292494 and GSE292495) and will be publicly released after the official acceptance of this manuscript.

## Supporting information

This article contains supporting information.

## Conflict of interest

The authors declare that they have no conflicts of interest with the contents of this article.
