# Supplementary material for: Macrophage-specific targeting of histone demethylases with small-molecule inhibitors suppresses inflammatory response in vivo
Source: J Biol Chem. 2026 Jul 6;302(8):113315. doi: 10.1016/j.jbc.2026.113315 (PMC13451880; doi:10.1016/j.jbc.2026.113315)
Supplement: Supplementary file 1 [file mmc1.docx]

**Supplementary figures and legends:**

**Figure S1: n-octyl-IOX1 and PBIT reduced *Mmp14, Ccl5*, and *Ccl7* in BMDM.** Inflammatory cytokine and *Mmp14* expression in BMDM treated with n-octyl-IOX1, PB-IT, GSK-J1, and GSK-J4 was quantified by qPCR. ** *p*<0.01, *** *p*<0.001, **** *p*<0.0001. one-way ANOVA with post-hoc Fisher LSD test. n=4/group.

**Figure S2: LPS treatment induces inflammatory genes in BMDM.** BMDM were treated with LPS, and RNA sequencing was performed. The volcano plot shows the differentially expressed genes in LPS-treated compared to control BMDM. FDR *p*<0.05, log2 fold change > 1. one-way ANOVA with post-hoc Fisher LSD test.

**Figure S3: n-octyl-IOX1 treatment reduces LPS-mediated inflammatory gene expression in BMDM.** BMDM were treated with LPS and cultured with or without n-octyl-IOX1, and RNA sequencing was performed. The volcano plot shows the differentially expressed genes in n-octyl-IOX1 compared to LPS-treated BMDM. FDR *p*<0.05, log2 fold change > 1.

**Figure S4: PB-IT treatment reduces LPS-mediated inflammatory genes in BMDM.** BMDM were treated with LPS and cultured with or without PB-IT, and RNA sequencing was performed. The volcano plot shows the differentially expressed genes in PB-IT compared to LPS-treated BMDM, FDR *p*<0.05, log2 fold change > 1.

**Figure S5**: **The distribution of most of the peaks was around the transcription start sites in LPS-exposed macrophages.** CUT&RUN for H3K4me3 and H3K9me3 was performed on murine BMDM pre-treated with n-octyl-IOX1 or PB-IT for 8 hours followed by LPS stimulation for 24 hours. Transcription start site plots for H3K4me3 and H3K9me3 are shown.

**Figure S6: n-octyl-IOX1 and PB-IT treatments reduce H3K4me3 and H3K9me3 motifs in the promoter regions.** CUT&RUN for H3K4me3 and H3K9me3 was performed on murine BMDM pre-treated with octyl-IOX1 or PB-IT for 8 hours followed by LPS stimulation for 24 hours. The summaries of the motifs differentially enriched in H3K4me3 (A) and H3K9me3 (B) are shown.

**Figure S7: n-octyl-IOX1 treatment does not alter the leukocyte frequencies in the blood, bone marrow, and spleen.** n-octyl-IOX1 (10 mg/kg) was administered into C57BL/6 mice intraperitoneally, followed by 1 mg/kg LPS injection after 24 hours. Leukocyte frequencies in the blood, bone marrow, and spleen were enumerated by flow cytometry. n=4/group. * *p*<0.05, ** *p*<0.01, 2-tailed Student’s t-test.

**Figure S8: Macrophage-specific delivery of n-octyl-IOX1 in mice treated with LPS. (**A&B) LNP size distribution was measured. DiO-labled n-octyl-IOX1 incorporated in lipidoid nanoparticles were injected intraperitoneally in female (C-E) and male (F) C57BL/6 mice before LPS injection. Empty LNP was injected as control. Leukocyte frequencies were evaluated by flow cytometry. n=10/group. * *p*<0.05, ** *p*<0.01, *** *p*<0.001, 2-tailed Student’s t-test.

**Figure S9: Macrophage-specific delivery of n-octyl-IOX1 in mice treated with LPS.** DiO-labled n-octyl-IOX1 incorporated in lipidoid nanoparticles was administered intraperitoneally into C57BL/6 mice before LPS injection. Empty LNP was injected as control. The expression of the inflammatory genes in sorted peritoneal monocytes (A), neutrophils (B), and B cells (C) was quantified. n=5/group. * *p*<0.05, 2-tailed Student’s t-test.
